# ﻿Distribution extension of a vent scale worm *Branchinotoglumabipapillata* (Polychaeta, Polynoidae) in the Indian Ocean

**DOI:** 10.3897/zookeys.1215.129623

**Published:** 2024-10-14

**Authors:** Won-Kyung Lee, Se-Joo Kim

**Affiliations:** 1 Division of Biomedical Research, Korea Research Institute Bioscience and Biotechnology, Daejeon 34141, Republic of Korea Division of Biomedical Research, Korea Research Institute Bioscience and Biotechnology Daejeon Republic of Korea; 2 Division of EcoScience, Ewha Womans University, Seoul 03760, Republic of Korea Ewha Womans University Seoul Republic of Korea; 3 KRIBB School, University of Science and Technology, Daejeon, 34113, Republic of Korea University of Science and Technology Daejeon Republic of Korea

**Keywords:** *16S* rRNA, *18S* rRNA, *CO1*, deep-sea, hydrothermal vent, northern Central Indian Ridge, polynoids

## Abstract

*Branchinotogluma* Pettibone, 1985 is the most species-rich genus within the subfamily Lepidonotopodinae Pettibone, 1983, comprising 18 valid species from chemosynthesis-based ecosystems in the Pacific and Indian Oceans. Here, we report a new distributional record of *Branchinotoglumabipapillata* Zhou, Wang, Zhang & Wang, 2018, at the hydrothermal vent sites on the northern Central Indian Ridge (nCIR). This record represents the northernmost occurrence of *B.bipapillata* in the Indian Ocean. We conducted a comparative study of the nCIR population and other documented populations using distributional information, morphological traits, and genetic markers (two mitochondrial [*COI*, *16S* rRNA] and one nuclear [*18S* rRNA] genes). While most morphological characters of *B.bipapillata* were consistent with those found in the Southwest Indian Ridge (SWIR), variations were noted in the segment with the last branchiae. Molecular data revealed that all populations of *B.bipapillata* form a single clade, indicating a wide distribution from the SWIR to nCIR, covering ~4,000 km across various ridges in the Indian Ocean. This study presents extensive distribution of a vent species with well-connected populations throughout the Indian Ocean, distinguishing it from many other vent species affected by the dispersal barrier in the Indian Ocean.

## ﻿Introduction

The subfamily Lepidonotopodinae Pettibone, 1983 consists of scale worms endemic to chemosynthesis-based ecosystems (Wu et al. 2023). Currently, seven species from the Indian Ocean are identified within this subfamily: three *Branchinotogluma*, two *Branchipolynoe*, and two *Levensteiniella* (Han et al. 2023). *Branchinotogluma* Pettibone, 1985, the most species-rich genus in the subfamily, comprises 18 species found in the Pacific and Indian Oceans (Han et al. 2023). Specifically, three *Branchinotogluma* species are distributed across different ridge systems in the Indian Ocean: *B.bipapillata*Zhou et al., 2018 in the Southwest Indian Ridge (SWIR) and southern Central Indian Ridge (sCIR), *B.jiaolongae*Han et al., 2023 in the SWIR and Carlsberg Ridge (CR), and *B.kaireiensis*Han et al., 2023 in the sCIR and CR (Zhou et al. 2018, 2022; Han et al. 2023).

Since the initial discovery of vent fields in the Indian Ocean in 2000, the Rodriguez Triple Junction, which links the SWIR, CIR, and Southeast Indian Ridge, was assumed to be a dispersal barrier for vent species within the Indian Ocean (Gamo et al. 2001; Chen et al. 2015). However, with the discovery of more vent fields and associated fauna, it now appears that the primary dispersal barriers lie within the ridge system itself, mainly due to ridge offsets, rather than between different ridge systems (Sun et al. 2020). For instance, the vent crab *Austinograearodriguezensis* Tsuchida & Hashimoto, 2002 was absent from the southern SWIR (sSWIR) but was found in the northern SWIR (nSWIR) and showed panmixia with populations from other ridges like the sCIR. Similarly, the distribution of the hairy snail *Alviniconcha* species complex shows connectivity between different ridges, northern CIR (nCIR) and CR, but subdivisions between the sCIR and nCIR on the same CIR (Sun et al. 2020; Jang et al. 2023).

While *B.bipapillata* has been reported from vent fields on two ridge systems, the SWIR and CIR, morphological and genetic studies were previously only conducted on specimens from the sSWIR. In this study, we collected *Branchinotogluma* species from hydrothermal vent fields on the nCIR and compared morphological and molecular data with those from vent fields on the sSWIR.

## ﻿Materials and methods

**Figure 1.**
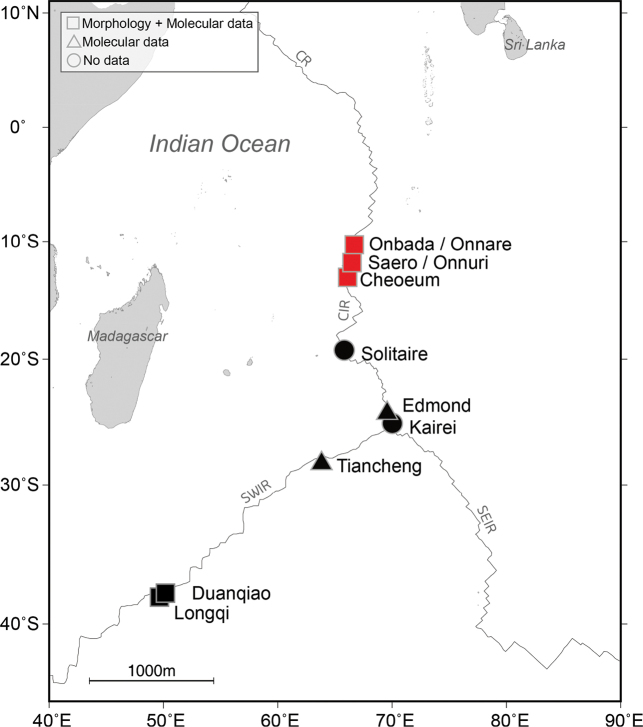
Map displaying the geographic distribution of *Branchinotoglumabipapillata* in the Indian Ocean. Red indicates sampling locations from this study and black indicates records of *B.bipapillata* from previous studies. Some closely situated sampling sites (< 10 km apart, such as Onbada and Onnare, Saero and Onnuri) are marked with a single square.

Specimens of *Branchinotogluma* were collected from hydrothermal vents in the nCIR during the 2023 KIOST expedition aboard the R/V *Isabu* (Fig. 1, Table 1) using a suction sampler and scoop mounted on the ROV ROPOS (Canadian Scientific Submersible Facility). Upon collection, a piece of elytron or parapodium from each specimen was dissected and preserved in 99% ethanol for molecular analysis. The entire body of the specimens was preserved in either 10% neutral buffered formalin or 70% ethanol for morphological studies.

**Table 1.** Sampling information of newly obtained *Branchinotoglumabipapillata* specimens from the nCIR and their GenBank accession numbers sequenced in this study.

**Voucher**	**Sampling site**	**Latitude (S), Longitude (E)**	**Depth (m)**	**GenBank Accession Numbers**		
				***CO1***	***16S***	***18S***
KRIBB310101–KRIBB310102	Cheoeum	12°37.1'S, 66°7.6'E	3018	PP600168–PP600169	PP600150–PP600151	PP600184– PP600185
KRIBB310103– KRIBB310107	Onnuri	11°24.9'S, 66°25.4'E	2009	PP600170– PP600174	PP600152– PP600156	PP600186– PP600190
KRIBB310108– KRIBB310110	Onnare	9°47.4'S, 66°41.9'E	2993	PP600175– PP600177	PP600157– PP600159	PP600191– PP600193
KRIBB310111– KRIBB310112	Onbada	9°48.9'S, 66°40.6'E	2563	PP600178– PP600179	PP600160– PP600161	PP600194– PP600195
KRIBB310113– KRIBB310116	Saero	11°19.7'S, 66°26.9'E	3256	PP600180– PP600183	PP600162– PP600165	PP600196– PP600199

For determination of morphological characters, all specimens were examined under a stereomicroscope (Stemi 508; Carl Zeiss, Germany). Specimen photographs were captured using a color camera (Axiocam 208 color; Carl Zeiss, Germany) and a DSLR camera (EOS 5D Mark IV; Canon, Tokyo, Japan). Images were processed with ZEN 3.3 blue edition (Carl Zeiss, Germany) and Helicon Focus software (Helicon Soft Ltd., Kharkov, Ukraine), and further edited using Adobe Photoshop 2022 (Adobe, San Jose, CA, USA). Specimen morphology was recorded following characters and states listed in Zhou et al. (2018).

A small piece of elytron or parapodium was used for total genomic DNA extraction using the AccuPrep® Genomic DNA Extraction Kit (Bioneer, Daejeon, South Korea), following the manufacturer’s instructions. Partial cytochrome c oxidase subunit 1 (*CO1*) and *18S* rRNA (*18S*) sequences were amplified following the protocols in Lee et al. (2021) and Jimi et al. (2021), respectively. For *16S* rRNA (*16S*), the primers 16SA (5′-CGCCGTTTATCAAAAACAT-3′) and 16Sbr (5′-CCGGTYTGAACTCAGATCAYG-3′) (Palumbi et al. 1991; Palumbi 1996) were used. Polymerase chain reaction (PCR) was conducted using a SimpliAmp™ Thermal Cycler (Applied Biosystems, Life technologies) under the following conditions: initial denaturation at 94 °C for 2 min; 5 cycles at 95 °C for 10 s, 42 °C for 30 s, and 72 °C for 60 s; 35 cycles at 95 °C for 10 s, 48 °C for 30 s, and 72 °C for 60 s; with a final extension at 72 °C for 2 min. PCR products were sent to Macrogen (Seoul, Korea) for Sanger sequencing.

New sequences were aligned with those of other Lepidonotopodinae species from GenBank (Suppl. material 1: table S1) using Geneious Prime ver. 2023.0.1 (Biomatters, Auckland, New Zealand). Sequence divergence for the *CO1* and *16S* genes was calculated using the *p*-distance method in MEGA11 (Tamura et al. 2021). For phylogenetic analysis, the three genes were concatenated using Geneious Prime. The best evolutionary model, GTR+I+G, was selected using jModelTest ver. 2.1.8 (Darriba et al. 2012). The phylogenetic tree was constructed using the maximum-likelihood method with raxmlGUI 2.0 (Edler et al. 2021).

All specimens used in this study are deposited at the Korea Research Institute of Bioscience and Biotechnology.

## ﻿Results

**Family Polynoidae Kinberg, 1856**

**Subfamily Lepidonotopodinae Pettibone, 1983**

### ﻿Genus Branchinotogluma

Taxon classificationAnimaliaPhyllodocidaPolynoidae

Pettibone, 1985

D20B503F-7044-5D35-86AE-3FE2A9BE060C

Branchinotogluma bipapillata Zhou, Wang, Zhang & Wang, 2018: 528–533, figs 1–7; table 1.

#### Material examined.

Indian Ocean • 2 ♂; Cheoeum; 12°37.1'S, 66°07.6'E; depth 3018 m; 28 Mar. 2023; W-K Lee leg.; hydrothermal vent; GenBank: PP600168– PP600169; KRIBB310101 to KRIBB310102 • 2 ♂, 2 ♀, 1 undetermined; Onnuri; 11°24.9'S, 66°25.4'E; depth 2009 m; 1–2 Apr. 2023; W-K Lee leg.; hydrothermal vent; GenBank: PP600170– PP600174; KRIBB310103 to KRIBB310107 • 1 ♀, 2 undetermined; Onnare; 9°47.4'S, 66°41.9'E; depth 2993 m; 3 Apr. 2023; W-K Lee leg.; hydrothermal vent; GenBank: PP600175– PP600177; KRIBB310108 to KRIBB310110 • 1 ♂, 1 ♀; Onbada; 9°48.9'S, 66°40.6'E; depth 2563 m; 4 Apr. 2023; W-K Lee leg.; hydrothermal vent; GenBank: PP600178– PP600179; KRIBB310111 to KRIBB310112 • 2 ♂, 2 ♀; Saero; 11°19.7'S, 66°26.9'E; depth 3256 m; 7 Apr. 2023; W-K Lee leg.; hydrothermal vent; GenBank: PP600180– PP600183; KRIBB310113 to KRIBB310116.

#### Description.

**Figure 2.**
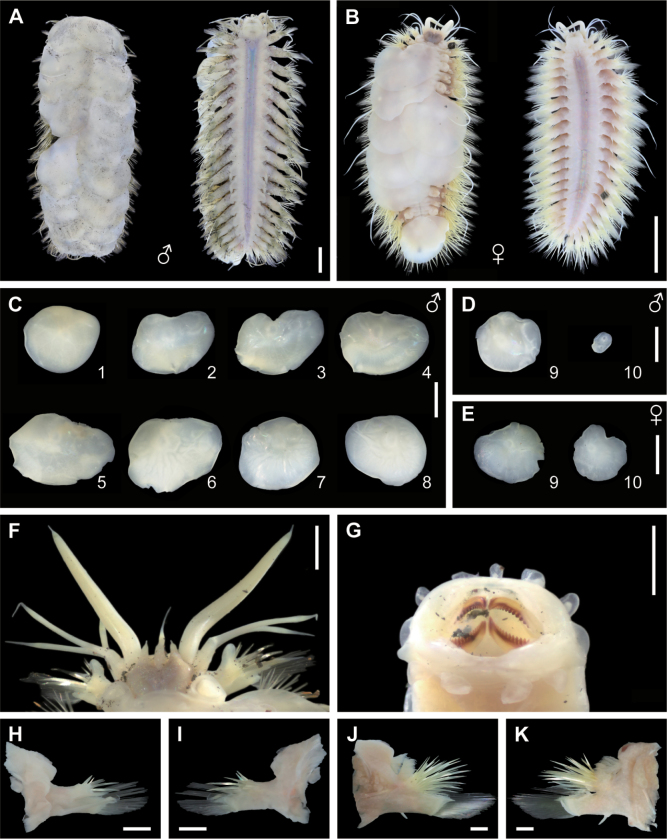
*Branchinotoglumabipapillata* specimens collected from the nCIR**A** dorsal and ventral views of male (KRIBB310116) **B** dorsal and ventral views of female (KRIBB310105) **C** 1^st^–8^th^ left elytra **D** 9^th^–10^th^ left elytra of male (KRIBB310103) E 9^th^–10^th^ left elytra of female (KRIBB310110) **F** head featuring prostomium, palps, tentacular cirri, and first parapodia on segment 2 (KRIBB310112) **G** everted pharynx with dorsal and ventral papillae (KRIBB310108). Anterior and posterior views of left parapodia on (H-I) segment 2 and (J-K) segment 11 (KRIBB310106). Scale bars: 5 mm (**A–E**); 0.5 mm (**F, G**); 1 mm (**H–K**).

Specimens relatively well preserved, with 21 segments, 12.0–51.0 mm in length and 5.0–16.6 mm in width. Body shape fusiform, tapered anteriorly and posteriorly (Fig. 2A, B, Table 2). Pairs of elytra on elytrophores on segments 2, 4, 5, 7, 9, 11, 13, 15, 17, and 19; elytra oval to subreniform, white, slightly transparent, with a smooth surface (Fig. 2C–E). Dorsal cirri on segments 3, 6, 8, 10, 12, 14, 16, 18, 20, and 21, extending beyond the tips of neurochaetae. Branchiae arborescent; grouped in two, one at base of the notopodia and another at base of dorsal tubercles or elytrophores; starting from segment 3 and ending between segments 18 or 21 (Table 2).

**Table 2.** Morphological comparison of *Branchinotoglumabipapillata* from the nCIR and sSWIR.

Region	Length (mm)	Sex (# of ind.)	Last segment with branchiae	Number of dorsal/ventral papillae on pharynx	9^th^ to 10^th^ elytrophore diatmeter ratio	Reference
**nCIR**	24.4–48.0	Male (5)	18	Not observed	2.25–2.64	This study
	20.5–51.0	Female (8)	18 or 21		1.08–1.46	
	12.0–17.8	Undetermined (3)	18	5/4*	1.23–1.28	
**SWIR**	23.3–32.3	Male (1)	18	5/5	N/A	Zhou et al. 2018
		Female (2)	19		N/A	

*Observed in a single specimen (KRIBB310108; Fig. 2G).

Prostomium bilobed, triangular anterior lobes with slender frontal filaments (Fig. 2F). Median antennae on anterior notch, with a cylindrical ceratophore and subulate style; palps thick, smooth, and end in subulate tips; lateral antennae and eyes absent (Fig. 2F). Tentacular segment fused to prostomium, with pair of tentacular cirri on each side, and a small acicular lobe at the base of tentaculophore; tentacular cirri slender (Fig. 2F).

First segment not distinct, fused to prostomium. Pharynx with five dorsal and four ventral papillae in one immature individual, but not seen in others (Fig. 2G). Second segment with first pair of elytrophores, ventral cirri, and biramous parapodia. Third segment with ventral cirri and first pair of branchiae. Fourth to last segments with ventral cirri and biramous parapodia. Notopodia smaller than neuropodia; notochaetae stout, few, arranged in radiating bundles; neurochaetae slender, numerous, forming a fan shape (Fig. 2H–K).

**Figure 3.**
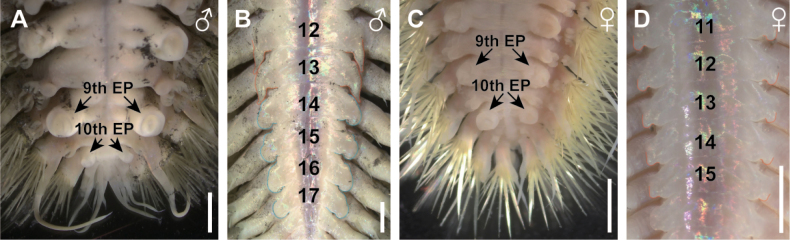
Sexually dimorphic characters of *Branchinotoglumabipapillata***A** dorsal view of posterior segments **B** ventral view of segments 12–17 of male (KRIBB310116) **C** dorsal view of posterior segments **D** ventral view of segments 11–15 of female (KRIBB310105). Arrows point to 9^th^ and 10^th^ elytrophores (EP) pointed with arrows. Ventral papillae are outlined in red and ventral lamellae in blue. Scale bars: 2 mm (A, C); 1 mm (B, D).

Sexual dimorphism evident. In males, posterior segments modified (Fig. 3A) with 10^th^ elytra and elytrophores much smaller than 9^th^ (Figs 2D, 3A, Table 2); ventral papillae present on segments 12–13, long, tapering, with slender tips extending to next segment; ventral lamellae on segments 14–17, round (Fig. 3B). In females, posterior segments not modified (Fig. 3C), with 10^th^ elytra and elytrophores similar to 9^th^ (Figs 2E, 3C, Table 2); ventral papillae present on segments 11–15, short and blunt (Fig. 3D).

#### Distribution.

Indian Ocean (depth 1732–3256 m): Longqi and Duanqiao vent fields on the southern Southwest Indian Ridge; Tiancheng vent field on the northern Southwest Indian Ridge; Edmond vent field on the southern Central Indian Ridge; Onnare, Onbada, Saero, Onnuri, and Cheoeum vent fields on the northern Central Indian Ridge.

#### Remarks.

Comparisons of key morphological characters between the geographically distant populations are present in Table 2. The key characters of the nCIR specimens of *B.bipapillata* largely correspond with those of the SWIR specimens (Zhang et al. 2018). However, the two populations differ in the last segment with branchiae in females (segment 19 in sSWIR compared with segment 18 or 21 in nCIR; Table 2).

Among the 16 specimens from the nCIR population, 10 specimens with body length greater than 20 mm were well-developed in all features indicating adult morphology, while characters of sexual dimorphism were not observed in 6 specimens shorter than 20 mm.

#### DNA barcoding and phylogenetic analysis.

Partial sequences of *CO1*, *16S*, and *18S* were recovered from 16 specimens collected from the nCIR. As shown in Table 1, 48 newly obtained sequences have been deposited in GenBank.

In *CO1*, the mean intra-population variation was 0.56% for nCIR and 0.65% for SWIR, with an inter-population variation of 1.00% (Table 3). In *16S*, the mean intra-population variation was 0.27% for nCIR and 0.33% for SWIR, with an inter-population variation of 0.39%. In *18S*, the mean intra-population variation was 0.01% for nCIR and 0.00% for SWIR, with an inter-population variation of 0.004%.

**Table 3.** Sequence divergence (%) among three *Branchinotoglumabipapillata* populations based on partial *CO1* gene (553 bp).

Populations	nCIR	sCIR	SWIR
(# of ind.; intra)			
**nCIR**	–		
(16; 0.56)			
**sCIR**	0.70	–	
(1; NC*)	(0.20–1.18)		
**SWIR**	1.00	0.47	–
(5; 0.65)	(0.00–1.65)	(0.20–1.19)	

*not calculated.

The interspecific variation between *B.bipapillata* and other congeners ranged from 18.63% to 21.88% in *CO1*, and from 13.11% to 19.08% in *16S* (Suppl. material 1: table S2). In *18S*, the interspecific variation ranged from 1.69% to 3.80%.

**Figure 4.**
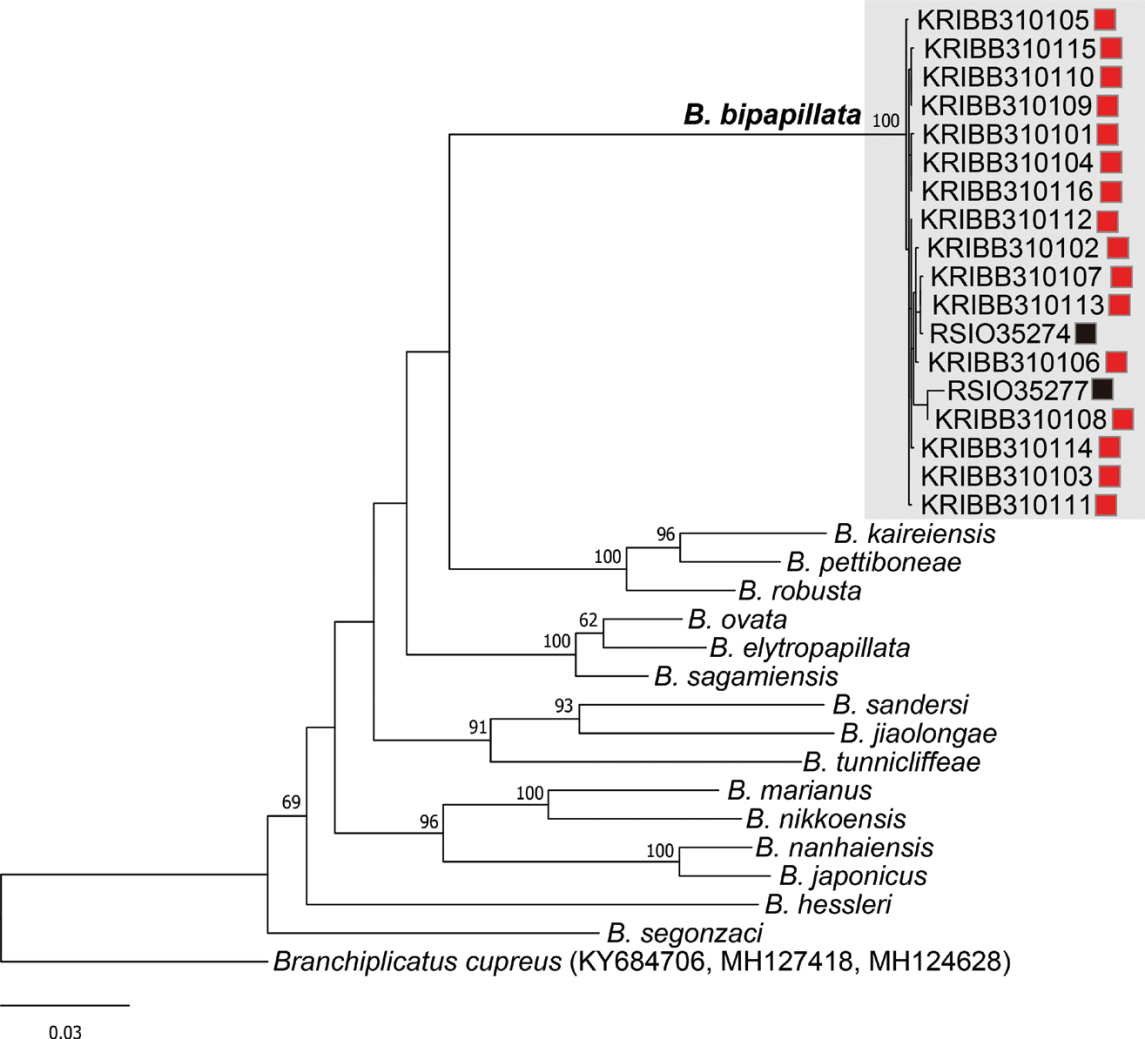
Maximum-likelihood phylogenetic tree of *Branchinotogluma* species based on concatenated sequences of the *CO1*, *16S*, and *18S* genes. *Branchinotoglumabipapillata* species are highlighted with a gray box. Red and black squares represent nCIR and sSWIR populations, respectively. GenBank accession numbers of the *CO1*, *16S* and *18S* genes of the outgroup are noted next to the species names. Maximum-likelihood bootstrap support values > 60 are displayed next to the nodes.

The maximum likelihood phylogenetic tree, constructed with concatenated sequences of *CO1*, *16S*, and *18S* (Fig. 4), shows the SWIR and nCIR populations of *B.bipapillata* clustering together as a single clade, indicating no significant divergence between populations from different ridges. Within the *Branchinotogluma* genus, *B.bipapillata* is closely related to a clade including *B.kaireiensis*, *B.pettiboneae*Wu et al., 2019, and *B.robusta*Wu et al., 2023.

## ﻿Discussion and conclusion

The vent scale worm *B.bipapillata* is widely distributed in the Indian Ocean, but comprehensive morphological and molecular data are lacking across all deep-sea oceanic ridges, and specimens are rarely reported at each sampling site (Fig. 1; only five specimens of *B.bipapillata* from the SWIR were barcoded with *CO1*, and only two sequences of *16S* and *18S* are available from SWIR specimens). In this study, 16 individuals, including female, male, and immature specimens of *B.bipapillata* from the nCIR were observed, enriching descriptions of features such as all elytra, and improving the molecular description of this species with both nuclear and mitochondrial gene sequences. Key morphological characters, such as the presence of an acicular lobe on the tentaculophore and the position of segmental ventral papillae, showed general congruence between the sSWIR and nCIR populations. Additionally, *CO1* barcode sequences revealed a mean intraspecific variation of 0.72% in *B.bipapillata*, which is within the variation range observed in other *Branchinotogluma* species (0.00–1.05%; Suppl. material 1: table S2).Thus, molecular data on genetic distances within and between populations showed no significant differences, and the phylogenetic analysis revealed a single clade of *B.bipapillata*, with no divergence between populations (Fig. 4, Table 3). Based on these morphological and molecular findings, the southernmost and northernmost populations appear to be well connected, forming a single genetic population with minimal morphological variability.

Other vent endemic species in the Indian Ocean, such as the mussel *Bathymodiolusmarisindicus* Hashimoto, 2001, the snail *Chrysomallonsquamiferum*Chen et al., 2015, the crab *A.rodriguezensis*, the barnacle *Neolepasmarisindica* Watanabe et al., 2018, and the worm *Ophryotrochajiaolongi* Zhang et al., 2017, all show a wide distribution range on the SWIR and CIR (Sun et al. 2020; Zhou et al. 2022). However, unlike the *B.bipapillata* populations in this study, most of these species exhibit low connectivity between populations, likely due to ridge offsets acting as dispersal barriers between the sSWIR and nSWIR, which do not seem to affect the connectivity of *B.bipapillata* (Sun et al. 2020; Zhou et al. 2022). Although the reproductive and larval development strategies of *B.bipapillata* are not fully understood, observations of other species within the same subfamily suggest that *B.bipapillata* likely have lecithotrophic larvae (Van Dover et al. 1999; Jollivet et al. 2000). This larval type, capable of traveling long distances in oligotrophic deep-sea environments, might partially explain the high connectivity of *B.bipapillata* populations across ~4,000 km of different ridges within the Indian Ocean.

Many studies have considered geological and hydrological features, along with the dispersal abilities of species, to explain the distribution of vent species (Slatkin 1987; Vrijenhoek 2010; Taylor and Roterman 2017; Perez et al. 2021). However, to fully understand the broad geographical distribution of these species, it is also crucial to consider their ability to adapt to diverse vent environments across different ridge systems. To further elucidate the strategies that enable species such as *B.bipapillata* to inhabit separate and geographically distant vent fields with no genetic differentiation, future studies should consider in vitro experiments for culturing as well as transcriptomic and genomic level data of populations.

## Supplementary Material

XML Treatment for Branchinotogluma

## ﻿Additional information

### Author ORCIDs

Won-Kyung Lee https://orcid.org/0000-0001-7283-298X

Se-Joo Kim https://orcid.org/0000-0003-1653-072X

### Data availability

All of the data that support the findings of this study are available in the main text or Supplementary Information.

## Supplementary materials

Supplementary material 1 Supplementary information Data type docx Explanation note **table S1.** Sample information and accession numbers of the *Branchinotogluma* species used in this study (new sequences are highlighted in bold). **table S2.** Interspecific divergence (%) of mitochondrial *CO1* (below left) and 16S (upper right) genes of *Branchinotogluma* species. Mean intraspecific *CO1* distances are displayed in bold along the diagonal. https://binary.pensoft.net/file/1153257This dataset is made available under the Open Database License (http://opendatacommons.org/licenses/odbl/1.0/). The Open Database License (ODbL) is a license agreement intended to allow users to freely share, modify, and use this Dataset while maintaining this same freedom for others, provided that the original source and author(s) are credited.Won-Kyung Lee, Se-Joo Kim
