# Supplementary material for: ﻿Distribution extension of a vent scale worm Branchinotoglumabipapillata (Polychaeta, Polynoidae) in the Indian Ocean
Source: Zookeys. 2024 Oct 14;1215:139–49. doi: 10.3897/zookeys.1215.129623 (PMC11494209; doi:10.3897/zookeys.1215.129623)
Supplement: Supplementary material 1 — Supplementary information [file zookeys-1215-139_article-129623__-s001.docx]

**Table S1.** Sample information and accession numbers of the *Branchinotogluma* species used in this study (new sequences are highlighted in bold).

| **Species** | **Voucher** | **Sampling site** | **Region, Ocean** | **Latitude** | **Longitude** | **Depth (m)** | **GenBank Accession Numbers** | | | | |
| --- | --- | --- | --- | --- | --- | --- | --- | --- | --- | --- | --- |
|  |  |  |  |  |  |  | ***CO1*** | ***16S*** | | | ***18S*** |
| *B. bipapillata* | KRIBB310101–KRIBB310102 | Cheoeum | nCIR, Indian | 12.62 °S | 66.13 °E | 3018 | **PP600168–PP600169** | **PP600150–PP600151** | | | **PP600184– PP600185** |
|  | KRIBB310103– KRIBB310107 | Onnuri | nCIR, Indian | 11.42 °S | 66.43 °E | 2009 | **PP600170– PP600174** | **PP600152– PP600156** | | | **PP600186– PP600190** |
|  | KRIBB310108– KRIBB310110 | Onnare | nCIR, Indian | 9.79 °S | 66.70 °E | 2993 | **PP600175– PP600177** | **PP600157– PP600159** | | | **PP600191– PP600193** |
|  | KRIBB310111– KRIBB310112 | Onbada | nCIR, Indian | 9.82 °S | 66.68 °E | 2563 | **PP600178– PP600179** | **PP600160– PP600161** | | | **PP600194– PP600195** |
|  | KRIBB310113– KRIBB310116 | Saero | nCIR, Indian | 11.33 °S | 66.45 °E | 3256 | **PP600180– PP600183** | **PP600162– PP600165** | | | **PP600196– PP600199** |
|  | RSIO35274  RSIO35277 | Longqi | sSWIR, Indian | 37.78 °S | 49.65 °E | 2761 | MH712481 MH712482 | MH717076  MH717077 | | | MH717080  MH717081 |
|  | CCIRN2 | Edmond | sCIR, Indian | 23.88 °S | 69.60 °E | n/a | OK267395 | n/a | | | n/a |
| *B. kaireiensis* | NSMT-Pol_P-922 | Kairei | sCIR, Indian | 25.32 °S | 70.04 °E | 2434 | OQ784744 | OQ785996 | | | OQ785977 |
| *B. jiaolongae* | RSIO35218 | Tiancheng | nSWIR, Indian | 27.95 °S | 63.53 °E | 2682 | OQ784749 | OQ785992 | | | OQ785976 |
| *B. trifurcus* | MBM286046 | n/a | Manus Basin, West Pacific | 3.73 °S | 151.67 °E | ~1800 | MK357905 | MK211415 | | | n/a |
| *B. segonzaci* | MBM286042 |  |  |  |  |  | MK357906 | MK211418 | | | MK211412 |
| *B. pettiboneae* | MBM286047 |  |  |  |  | 1707 | OL703004 | OL699915 | | | OL699912 |
| *B. ovata* | MBM286062-sps2 |  |  | 3.68 °S | 151.86 °E | 1853 | MK357896 | MK211416 | | | MK211411 |
| *B. marianus* | SIO:BIC:A13116 | n/a | North Fiji Basin, West Pacific | n/a | n/a | 1990 | MW646931 | MW654556 | | | MW654525 |
| *B. nikkoensis* | NSMT-Pol H-880 | n/a | Nikko Seamount, West Pacific | 23.08 °N | 142.33 °E | 458 | ON255504 | ON244617 | | | ON244619 |
| *B. elytropapillata* | NSMT-pol P-668 | Sakai | West Pacific | 27.55 °N | 126.99 °E | 1286 | MG799387 | MG799377 | | | MG799378 |
| *B. japonicus* | - |  |  | n/a | n/a | n/a | KY753824 | KY753824 | | | KY753841 |
| *B. sagamiensis* | NSMT-Pol H-885 | Off Hatsushima | Sagami Bay, West Pacific | 36.02 °N | 139.22 °E | 867 | ON255503 | ON244616 | | | ON244618 |
| *B. robusta* | MBM286805-sps2 | Seep | South China Sea, West Pacific | 22.12 °N | 119.29 °E | 1119 | OL703005 | OL699916 | | | OL699913 |
| *B. nanhaiensis* | MBM286800-sps4 |  |  |  |  |  | OL702996 | OL699914 | | | OL699911 |
| *B. tunnicliffeae* | SIO:BIC:A7717 | n/a | Juan de Fuca Ridge, East Pacific | n/a | n/a | 2250 | MW646935 | | MW654560 | MW654530 | |
| *B. sandersi* | SIO:BIC:A6321 | n/a | Southern Gulf of California, East Pacific | n/a | n/a | n/a | KY684716 | | MH127416 | MH124627 | |
| *B. hessleri* | SIO:BIC:A6316 |  |  | 23.36 °N | 108.53 °W | 2299 | KY684713 | | MH127414 | MH124626 | |

**Table S2.** Interspecific divergence (%) of mitochondrial *CO1* (below left) and *16S* (upper right) genes of *Branchinotogluma* species. Mean intraspecific *CO1* distances are displayed in bold along the diagonal.

| **Species (# of inds.)** | **1** | **2** | **3** | **4** | **5** | **6** | **7** | **8** | **9** | **10** | **11** | **12** | **13** | **14** | **15** | **16** | **17** |
| --- | --- | --- | --- | --- | --- | --- | --- | --- | --- | --- | --- | --- | --- | --- | --- | --- | --- |
| **1. *B. bipapillata* (22)** | **0.72** | 16.07 | 15.36 | 16.50 | 15.36 | 16.67 | 18.63 | 15.08 | 15.64 | 13.11 | 14.98 | 19.08 | 15.79 | 15.36 | 15.03 | 15.36 | 18.30 |
| **2. *B. trifurcus* (1)** | 18.63 | **-** | 15.58 | 13.86 | 12.79 | 14.10 | 14.75 | 7.24 | 16.61 | 6.91 | 11.76 | 12.42 | 12.13 | 15.36 | 16.61 | 17.32 | 17.97 |
| **3. *B. tunnicliffeae* (1)** | 19.17 | 19.71 | **-** | 15.79 | 16.61 | 14.66 | 15.31 | 14.38 | 17.53 | 14.38 | 16.23 | 17.26 | 17.92 | 14.56 | 17.26 | 17.26 | 17.26 |
| **4*. B. nikkoensis* (1)** | 19.35 | 19.35 | 21.70 | **-** | 13.53 | 10.82 | 18.36 | 14.47 | 15.84 | 12.17 | 15.13 | 17.05 | 15.18 | 17.43 | 17.16 | 17.88 | 17.82 |
| **5. *B. elytropapillata* (4)** | 19.53 | 20.61 | 19.17 | 20.43 | **0.00** | 14.33 | 14.38 | 12.66 | 12.46 | 13.36 | 4.85 | 15.74 | 4.58 | 13.40 | 13.49 | 13.16 | 17.05 |
| **6*. B.* cf. *marianus* (1)** | 19.71 | 17.72 | 21.34 | 14.83 | 20.80 | **-** | 16.29 | 13.96 | 14.71 | 13.59 | 13.31 | 15.31 | 14.75 | 14.75 | 15.08 | 15.74 | 17.76 |
| **7. *B. jiaolongae* (3)** | 19.71 | 17.90 | 17.72 | 17.72 | 20.43 | 18.63 | **0.45** | 15.03 | 18.24 | 14.05 | 14.98 | 18.57 | 14.38 | 13.03 | 17.97 | 18.30 | 16.99 |
| **8. *B. nanhaiensis* (8)** | 19.71 | 20.98 | 20.61 | 18.26 | 21.16 | 18.99 | 20.98 | **0.04** | 13.49 | 5.21 | 12.34 | 10.49 | 11.80 | 13.44 | 14.19 | 13.20 | 17.43 |
| **9. *B. robusta* (12)** | 19.89 | 18.08 | 20.43 | 21.16 | 19.53 | 19.53 | 19.35 | 20.07 | **0.42** | 13.77 | 12.75 | 18.24 | 14.05 | 16.34 | 5.16 | 5.83 | 20.92 |
| **10. *B. japonicus* (3)** | 20.25 | 22.24 | 21.88 | 18.26 | 22.06 | 18.63 | 20.07 | 8.32 | 21.70 | **0.24** | 13.36 | 12.09 | 12.83 | 14.14 | 14.80 | 15.13 | 15.84 |
| **11*. B. sagamiensis* (1)** | 20.25 | 18.44 | 19.35 | 19.35 | 9.58 | 16.82 | 18.81 | 19.17 | 19.35 | 20.61 | **-** | 14.71 | 4.23 | 14.66 | 12.79 | 13.77 | 16.34 |
| **12. *B. segonzaci* (12)** | 20.43 | 18.99 | 20.80 | 19.35 | 17.90 | 18.81 | 18.26 | 16.27 | 20.61 | 17.00 | 14.65 | **0.38** | 15.08 | 16.07 | 17.92 | 17.70 | 17.76 |
| **13. *B. ovata* (6)** | 20.80 | 19.53 | 19.71 | 18.99 | 7.96 | 18.08 | 18.99 | 18.99 | 19.71 | 20.61 | 7.78 | 17.36 | **0.14** | 14.05 | 14.10 | 15.03 | 17.38 |
| **14. *B. sandersi* (8)** | 20.80 | 17.72 | 19.17 | 19.53 | 19.71 | 19.17 | 15.73 | 20.07 | 20.98 | 23.15 | 18.81 | 19.17 | 17.90 | **0.04** | 17.05 | 18.03 | 17.48 |
| **15. *B. kaireiensis* (4)** | 21.52 | 17.72 | 21.52 | 20.98 | 20.80 | 17.90 | 19.71 | 21.88 | 13.20 | 22.24 | 19.89 | 21.52 | 19.35 | 20.61 | **1.05** | 5.19 | 20.33 |
| **16. *B. pettiboneae* (5)** | 21.70 | 20.43 | 22.97 | 20.80 | 19.17 | 20.07 | 20.43 | 22.06 | 13.02 | 22.24 | 19.53 | 21.52 | 18.99 | 20.43 | 11.03 | **0.07** | 21.31 |
| **17. *B. hessleri* (1)** | 21.88 | 18.63 | 21.16 | 21.52 | 17.72 | 20.80 | 20.25 | 18.99 | 17.00 | 20.43 | 18.44 | 20.07 | 19.71 | 19.35 | 20.07 | 20.80 | - |
